# Die ordnungsstiftende Funktion pädagogischer Technologien und Kernaktivitäten in der Erwachsenenbildung. Relationierung zweier qualitativer Forschungsperspektiven

**DOI:** 10.1007/s40955-022-00202-0

**Published:** 2022-03-29

**Authors:** Jenny Kipper, Dieter Nittel

**Affiliations:** 1 Goethe-Universität Frankfurt a. M., Frankfurt a. M., Deutschland; 2 Fernuniversität Hagen, Hagen, Deutschland

**Keywords:** Didaktik, Pädagogisches Handeln, Pädagogische Technologien, Kernaktivitäten, Erwachsenenbildung, Didactic, Educational action, Educational technologies, Key activities, Adult education

## Abstract

Das aus der Sozialen-Welt-Theorie von A. L. Strauss stammende Konzept „pädagogische Technologien und Kernaktivitäten“ wurde bislang vorrangig auf grundlagentheoretische pädagogische Fragestellungen angewendet. Der vorliegende Beitrag überträgt dieses Konzept erstmalig auf die Erwachsenenbildung. Die Autoren unterstreichen in dem Beitrag, dass abduktive und relationale Verfahren geeignet sind, zwischen dem Mikrobereich der symbolisch vermittelten Interaktion in der Erwachsenenbildung und der Ebene des pädagogisch organisierten Systems des lebenslangen Lernens Verbindungen herzustellen.

## Abstract (en)

The concept of technologies and key actions, based on A. L. Strauss’ social-world theory, has mainly been applied to substantial theoretical pedagogic questions. The present contribution transfers this concept for the first time on adult education. The authors emphasise in their article that abductive and relational techniques are applicable to connect the microfield of symbolically conveyed interaction in adult education with the level of the pedagogically organised system of lifelong learning.

## Das Konzept Pädagogische Technologien und Kernaktivitäten – im Horizont abduktiver Forschung

Das Spektrum an Konzepten, um die Vielfalt pädagogischer Handlungsformen im Erziehungs- und Bildungswesen zu systematisieren, ist in der Erziehungswissenschaft recht überschaubar. Zwei Ansätze gilt es an dieser Stelle exemplarisch zu würdigen: So nimmt das Modell von Giesecke (1997) eine Schlüsselstellung ein, weil es gleich in mehreren erziehungswissenschaftlichen Subdisziplinen auf starkes Interesse stieß. Auf der Grundlage des Befundes, dass sich intentionales pädagogisches Handeln in organisierten Settings mittlerweile auf den gesamten Lebenslauf erstrecke, geht es ihm um nichts Geringeres als um eine erste Skizze einer Rahmentheorie pädagogischer Berufe. Mit der Beschreibung der Grundformen des pädagogischen Handelns (Unterrichten, Organisieren, Arrangieren, Informieren und Beraten) versucht Giesecke die Bauformen pädagogischen Handelns zu erfassen und somit den Boden für eine möglichst divers konfigurierte berufliche Identität von Pädagogen zu bereiten. Eine ähnliche Absicht verfolgen Prange und Strobel-Eisele (2006). Auch sie liefern ein Ordnungsschema zur Erschließung des pädagogischen Handelns in prinzipiell allen Feldern der Erziehung und Bildung. Eine strategisch wichtige Rolle spielt dabei das Zeigen, wobei zwischen vier verschiedenen Formen des Zeigens differenziert wird. Neben den elementaren Formen des pädagogischen Handelns werden komplexe Formen benannt (das Arrangement, das Spiel, die Arbeit, das Erlebnis und die Strafe) sowie makrodidaktische Ebenen erörtert und Großformen pädagogischen Handelns (Volks- und Massenerziehung, Umerziehung, Erziehung durch Medien) erläutert.

Das von der komparativen pädagogischen Berufsgruppenforschung (Nittel et al. 2014; Meyer 2017; Wahl 2017; Schütz 2018; Nittel und Tippelt 2019) eingeführte Konzept der pädagogischen Technologien und Kernaktivitäten erhebt einen ähnlichen Anspruch wie die beiden erwähnten Modelle. Im Unterschied zu den Systematiken von Giesecke und Prange/Strobel-Eisele beruht die in diesem Beitrag vorgestellte Systematik nicht nur auf Theoriearbeit, sondern auch auf empirischer Forschung und auf einem längeren Prozess der abduktiven Erkenntnisgewinnung.^1^ Im Rahmen der PAELL-Studie^2^ (Nittel et al. 2014) sind die pädagogischen Technologien (Programme, Arbeits- und Veranstaltungsformen, Methoden und Medien) und die Kernaktivitäten (des Unterrichtens, Begleitens, Beratens, Sanktionierens und Organisierens) in einem ersten Schritt induktiv entwickelt worden. Die Datengrundlage dieses Entdeckungszusammenhangs bestand aus offenen Antworten von 1601 Fragebögen und den Transkriptionen aus 27 Gruppendiskussionen (Nittel et al. 2014). Eine Verifizierung und Konsolidierung der Schlüsselkategorien der pädagogischen Technologie erfolgte in einer rekonstruktiv angelegten Dissertation über das pädagogische Handeln von Vertretern der betrieblichen Bildung (Kipper 2014).^3^ Im Zuge eines zweiten Schritts wurden im Rahmen eines universitären Lehrforschungsprojektes auf der Grundlage des angedeuteten Inventars an Kategorien Fachkräfte aus allen Segmenten des pädagogisch organisierten Systems des lebenslangen Lernens mittels Kurzinterviews von Studierenden nach ihren Alltagspraktiken befragt. Das hypothesenüberprüfende Vorgehen folgte einer deduktiven Logik. Der zentrale Befund aus den mehr als tausend Kurzinterviews lautet, dass das Unterrichten, das Beraten, das Begleiten, das Sanktionieren und das Organisieren keine künstlichen, sondern vertraute Sprachspiele sind, mit denen sich Fachkräfte in pädagogischen Handlungsfeldern im Sinne von In-Vivo-Codes (Strauss 1998) verständigen. Dieser Schritt der Verifizierung und Konsolidierung wurde zum einem in einem grundlagentheoretischen Beitrag (Nittel et al. 2020) und zum anderen in einer vertiefenden, induktiv angelegten Teilstudie über die Kernaktivität des Begleitens (Nittel und Meyer 2018) abgeschlossen. Auch die Edition eines Handbuchs zur Beratung kann dieser Phase zugerechnet werden (Gieseke und Nittel 2016).

Die vorliegende Publikation markiert den dritten Schritt. Bislang konnte in dieser Sequenz am Beispiel der Schule (vgl. Nittel 2021) dargestellt werden, wie durch das geteilte Wissen über die pädagogischen Technologien und Kernaktivitäten das Wir-Gefühl und die Solidarität in der Lehrerschaft gestärkt werden könnte. Ein anderer Beitrag für die Elementarpädagogik (Buschle und Nittel 2022) nutzt die auch hier bemühten Kernkategorien und Dimensionen, um die offene Frage der Systemzugehörigkeit der vorschulischen Bildung zu klären. Was haben die Leserinnen und Leser im vorliegenden Beitrag zu erwarten? In diesem Beitrag soll das Modell auf die Erwachsenenbildung appliziert werden. Damit wird aber nicht nur die Tragfähigkeit des Konzepts im Bereich Weiterbildung unterstrichen, sondern der Ansatz zugleich auch in der Weise weiterentwickelt, dass die Relation der Technologien zu den Kernaktivitäten transparenter wird. Während alle Publikationen aus dem Umkreis der komparativen pädagogischen Berufsgruppenforschung die Beziehung von Technologie und Kernaktivität bislang analog zu dem Verhältnis von situativen/habitualisierten Praktiken einerseits und institutionalisierten Vermittlungsformaten andererseits modelliert haben, ohne die Brücke zwischen beiden Bereichen aufzeigen zu können, schlägt dieser Text einen neuen Weg ein. Mit der methodischen Kombination soll auch eine gegenstandsorientierte Relationierung angestoßen werden. Für den vorliegenden Artikel diente exemplarische Erwachsenenbildungsliteratur – speziell Trainer- und Methodenliteratur – als Basis einer Dokumentenanalyse.^4^ Dabei handelt es sich um 17 Werke, die in erster Linie der sogenannten Methodenliteratur^5^ der Erwachsenenbildung angehören und den Methodendiskurs und dessen Verwendung exemplarisch widerspiegeln.^6^ Auf Basis der vorausgehend erstellten Definitionen im Stil der Qualitativen Inhaltsanalyse nach Mayring (2004) erfolgt eine Analyse der Schriften und eine Zuordnung der darin erwähnten Technologien. Der Vorteil dieses deduktiv geprägten Vorgehens ist, dass wir die Semantik auf der Ebene der Methoden und des didaktischen Handwerkszeugs mit dem von uns entwickelten Kategoriensystem abgleichen können, ohne in den Fehler zu verfallen, das Eine gegenüber dem Anderen als besser oder schlechter zu qualifizieren. Im nächsten Abschnitt erfolgt ein exemplarischer Einblick in die Praxis der Erwachsenenbildung, wobei sich die Aussagen auf einer ähnlich konkreten Ebene bewegen wie in der Dokumentenanalyse. Die zunächst profan wirkenden quasi-ethnographischen Impressionen aus der Praxis der Erwachsenenbildung erheben keineswegs den Anspruch einer Realanalyse. Vielmehr liefern sie Anknüpfungspunkte, um die Leichtigkeit und Mühelosigkeit zu demonstrieren, mit der die Dimensionen der pädagogischen Kernaktivitäten des Organisierens, Begleitens, Beratens und Unterrichtens im Alltag einer Volkshochschule identifiziert werden können. Die Kombination eines etablierten mit einem eher unkonventionellen Forschungszugangs bereitet dann schließlich den Boden, um in einem offenen Suchprozess die bislang noch nicht ins Werk gesetzte Relationierung von Technologien und Kernaktivitäten praktisch zu vollziehen. Dem erwachsenenpädagogischen Publikum liefert dieser abduktive Schritt die eine oder andere Überraschung. Immerhin wird hier nicht nur die Verschränkung von Organisation/Administration/Bürokratie einerseits und Professionalität/pädagogischer Fachlichkeit andererseits transparenter, sondern es kann auch eine zentrale, bisher unerkannte Dimension in der Beziehung von Organisations- und Systemebene aufgedeckt werden. Die Befunde im letzten Abschnitt standen zu Beginn des Schreibprozesses an diesem Beitrag keineswegs fest, sondern haben sich erst im Zuge des Umgangs mit den Irritationen ergeben, welche die Kontrastierung der Befunde aus einer Dokumentenanalyse mit den konkreten Phänomenen eines quasi-ethnographischen Bericht ausgelöst haben. Analog zu dem modernen Verständnis, dass sich der abduktive Schluss nicht nur als blitzartige Überraschung darbietet, haben wir in diesem Beitrag an der Annullierung der Überraschung durch das Aufstellen einer neuen Regel gearbeitet.

## Pädagogische Technologien

Unter dem Begriff der pädagogischen Technologie fassen wir die Gesamtheit soziokultureller, infrastruktureller und personeller Mittel, die notwendig sind, um organisiertes Lehren und Lernen in Erziehungs- und Bildungseinrichtungen – in diesem Fall: der Erwachsenenbildung/Weiterbildung – möglich zu machen. Wir verwenden den Begriff *Technologie* nicht im Sinne von Luhmann und Schorr (1982), sondern angelehnt an die Tradition des Symbolischen Interaktionismus und die Theorie sozialer Welten (vgl. Strauss 1990). Pädagogische Technologien beschreiben eine Form der Ordnung: „There is a technologicial order, easily seen if one thinks of action that requires machinery of equipment or other ‚hard‘ technology; but technical order is equally characteristic of any kind of action – there are always at least procedures that order pertaining significant ‚soft‘ technology“ (Strauss 1993, S. 59). Technologie darf somit nicht auf Technik im Sinne von *hard-ware* reduziert werden. In unserem Verständnis richten wir uns am antiken Ursprung des Wortes Technologie aus, also an einer vom praktischen Tun losgelösten *Philosophie* vom Handwerk (vgl. Beckmann 1777). Der Begriff inkludiert im Weiterbildungszusammenhang die Totalität der von der Organisation bereitgestellten Ressourcen, die im Sinne einer sachlich, zeitlich, räumlich und personell überformten Zweck-Mittel-Relation (vgl. Ropohl 2009, S. 21 f.) der pädagogischen Arbeit Arenen zu ihrer Entfaltung bieten. Konstitutiv für Technologie im Sinne einer Sozialtechnologie ist, dass sie von denen, die sie verwenden, mit der Erwartung eines festen Wirkungszusammenhangs verknüpft wird. Wir unterscheiden zwischen vier Technologien: *Programmen, Arbeits- und Veranstaltungsformen, Methoden* und *Medien*. Programme verknüpfen lebenszyklische Prozesse der Teilnehmenden (Entwicklungen, Übergänge und Lernbedarfe im Erwachsenenalter) mit dem Zeittakt der Organisation. Arbeits- und Veranstaltungsformen verbinden die Alltagszeit pädagogischer Organisationen mit dem Zeitrhythmus der Teilnehmenden in ihrer Lebenspraxis. Methoden und Medien bringen die situativen Vermittlungsdimension der pädagogischen Kommunikation im Hier und Jetzt mit den Aneignungspraktiken der Teilnehmenden zusammen. Technologien spannen somit den Bogen zwischen dem Zeithorizont institutioneller Strukturen im Makromaßstab und der singulären pädagogischen Situation im Mikrobereich.

### Programme

Programme sind teils schriftlich fixierte, teils mündlich tradierte Lehr-Lernarrangements, die durch ihre organisatorischen, technischen, zeitlichen und räumlichen Komponenten das Angebot einer pädagogischen Organisation in Gänze abbilden (vgl. Kipper 2014, S. 161 f.). Sie orientieren sich am Lebenslauf und fußen auf einem gesellschaftlichen Mandat, ein Individuum in einem Abschnitt des Lebenszyklus zu begleiten bzw. von einem Übergang zum nächsten Übergang zu führen. So gesehen weisen Programme eine große Ähnlichkeit mit jenen institutionellen Ablauf- und Erwartungsmustern des Lebenslaufs (Schütze 1981; Nittel 2017) auf, die sich auf organisierte Erziehungs- und Bildungsmaßnahmen beziehen und über das sich die durchführende/anbietende Organisation identifiziert (vgl. Nittel et al. 2020, S. 386 ff.).

Programme entstehen durch die Verdichtung verschiedener Arbeits- und Veranstaltungsformen (Programmplanung; vgl. Schrader 2012, S. 35). Die ausführende Organisation setzt die in einem Programm enthaltenen Maßnahmen und die darin gebündelten Arbeits- und Veranstaltungsformen um und rahmt diese durch standardisierte Rituale ein. Durch die Lebenslaufbezogenheit steigt ihre Varianz mit zunehmender Alters- und Teilnehmendenstruktur. In der Erwachsenenbildung erreichen Programme ihre größtmögliche Vielfalt im Rahmen formaler und non-formaler Bildungsangebote.

Programme können in ihrer Länge variieren. So gibt es in der Erwachsenenbildung einerseits zeitlich stark begrenzte Programme, wie den einmaligen Töpferkurs an der Volkshochschule und andererseits langjährige Formate, wie z. B. die mehrjährige Coaching- oder Trainerausbildungen bei privatwirtschaftlichen Anbietern oder die Vorbereitungs‑, Grundlagen- und Aufbaukurse beim Cambridge Zertifikat.

Charakteristisch für Programme sind die mit ihnen verbundenen Rituale. So gibt es Einstiegs- und Ausstiegsrituale, die in Teilen der Erwachsenenbildung auch durch einen formalen Akt der Organisation ersetzt werden können. So laufen z. B. Einschreibeprozesse heutzutage hochstandardisiert und automatisiert ab, wohingegen in der Vergangenheit das Einschreiberitual aus einem analogen Prozess inklusive Beratungsgespräch bestand. Ein Ausstiegsritual kann ein formaler Akt der Verabschiedung sein, die Übergabe eines Zertifikats, mindestens jedoch einer Teilnahmebescheinigung.^7^

**Figure 1:**
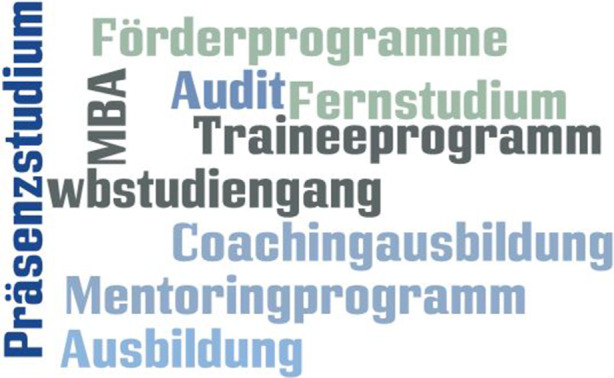

Programme können entweder sequenziell anschlussfähig sein oder eine separate Funktion erfüllen. In der betrieblichen Bildung dient beispielsweise das Traineeprogramm als Basis für ein Mentoring- oder Nachwuchsführungsprogramm; die Coachingausbildung ersetzt Teile der Organisationsentwicklungsausbildung. Die spezifische Form von Programmen, so zeigen es die Daten, kann auf Lernformate in Präsenz ausgerichtet sein oder Selbstlernen in den Vordergrund stellen – so z. B. im Kontext des Fernstudiums (vgl. Götz und Häfner 1994, S. 80 ff.). Der Fokus auf das Selbstlernen ist auch bei Frey (1995) ersichtlich. Er beschreibt Möglichkeiten, Projektarbeit im Lernalltag von Schülerinnen und Schülern großflächig zu verankern und führt z. B. das Programm „Lehrplan“ (Frey 1995, S. 173 ff.) als makrodidaktisches Element an, in dem Räume für Projektarbeit geschaffen werden können. Herzer et al. (1997) befassen sich mit dem Projektlernen in der Ausbildung: Innerhalb von Programmen (in diesem Fall dem Lehrplan) werden hier die Arbeits- und Veranstaltungsformen strukturiert. Da Programme in unserer Literaturbasis eher weniger präsent waren, haben wir die nachfolgende Darstellung mit Beispielen angereichert, die wir aus Quellen jenseits der Methodenliteratur bezogen haben^8^ (Abb. 1).

Der Umstand, dass in der zugrunde liegenden Datenbasis keine Definition für Programme gefunden werden konnte, liegt daran, dass Programme gewöhnlich strikt von Methoden getrennt werden. Lediglich ein Anhaltspunkt findet sich bei Götz und Tschacher (1995), wenn sie von Lernprogrammen (ebd., S. 15) sprechen. Insgesamt legt das Material die Deutung nahe, dass die Zuordnung der Technologien stark autoren- und perspektivenabhängig ist und sich nicht an allgemeingültigen Definitionen orientiert. Die starke Fixierung auf nur eine Technologiekategorie kann dazu führen, dass ein differenzierter Blick auf die sachlogischen Unterschiede der Technologien tendenziell verloren geht. So spricht Schäffter (1994) den auch aus unserer Sicht relevanten Punkt an, dass eine kommunikative Herausforderung zwischen pädagogisch Tätigen oftmals daraus resultiert, dass aus einer didaktischen Sicht der Verzahnung der Technologien zu wenig Aufmerksamkeit beigemessen wird (ebd., S. 21 ff.).^9^

Zusammenfassend können wir festhalten, dass von der Organisation vorgegebene Programme die Summe aller Angebote im Sinne von *People processing strategies of organization socialisation* enthalten, in ihrer Länge variabel sind und durch Ein- und Ausstiegsprozeduren gerahmt werden. Aktuell lässt sich vermehrt eine Tendenz zu kleinschrittigeren Programmen feststellen. Sie sind streng auf ihre pädagogische Zielgruppe ausgerichtet, begleiten institutionelle Ablauf- und Erwartungsmuster und fokussieren sowohl auf angeleitetes Lernen als auch Selbstlernen. Programme greifen auf ein formalisiertes Curriculum, welches das offizielle Mandat der Einrichtung widerspiegelt, zurück und grenzen sich dadurch von Arbeits- und Veranstaltungsformen ab.

### Arbeits- und Veranstaltungsformen

**Figure 2:**
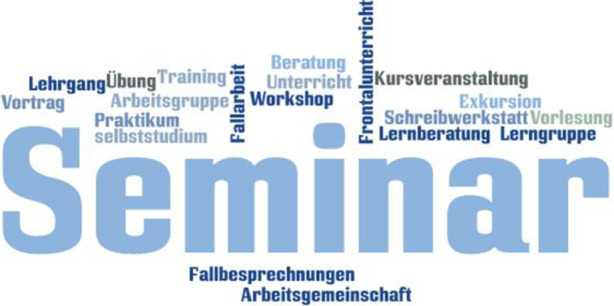

Ein Programm kombiniert in der Regel mehrere Arbeits- und Veranstaltungsformen (Abb. 2) und die Individualität des Programms leitet sich maßgeblich aus deren Verschränkung mit den übrigen Technologien ab. Arbeits- und Veranstaltungsformen sind, genauso wie die Programme, von der Organisation vorgegeben und in der Konzeption von der Einrichtung oder anderer Stelle schriftlich kodifiziert. Der pädagogisch Handelnde verfügt über die Autonomie, sie mit Leben, sprich mit Methoden und Medien, zu füllen. Er agiert damit als Verfahrensverwalter der darin „stattfindenden Vermittlungs- und Aneignungsprozesse“ (Kipper 2014, S. 161). Durch die Arbeits- und Veranstaltungsform wird er zeitlich und räumlich begrenzt, gleichzeitig geben sie Spielraum und Möglichkeiten des pädagogischen Settings vor. Man kann sie damit auch als Taktgeber für den pädagogischen Alltag (vgl. Nittel et al. 2020, S. 388) bezeichnen, die den Routinebetrieb im Spektrum zwischen einer Woche und einem Tag bestimmen.

Der Begriff „Veranstaltungs- und Arbeitsform“ (oder „Aktionsform“) wird in der untersuchten Literatur vielfach verwendet, allerdings mit gewissen Redundanzen zur Methode. Schäffter (1994, S. 53 ff.) subsumiert unter „Grundformen des Lehrens und Lernens“ (ebd., S. 5) sowohl Arbeits- und Veranstaltungsformen als auch Methoden und unterteilt diese in ihre Wirkungszonen (Gesamtwirkung, Außenwirkung, Einwirkung und Binnenwirkung; vgl. ebd., S. 59). Ähnliches findet sich auch bei Siebert (2004). Knoll definiert die Veranstaltungsform als „den jeweiligen Rahmen, innerhalb dessen sich Menschen bei einem Angebot der Erwachsenenbildung treffen“ und der für die „Arbeitsbeziehung zwischen Leitung und Teilnehmenden“ und den „Austausch zwischen den Teilnehmenden“ (Knoll 1999, S. 75) gestaltend ist. Götz und Häfner sprechen von „Organisationsformen“ und definieren diese Kategorie in einer ähnlichen Weise wie das hier vorgestellte Konzept der Arbeits- und Veranstaltungsformen (Götz und Häfner 1994, S. 80). Diese gliedern die Organisationsformen in „Klassenzimmer“, „Übergangsformen“ und „Selbststudiumsformen“ (ebd., S. 80). Schäffter schlägt zur Systematisierung von Veranstaltungsformen eine Differenzierung nach zeitlicher, sachlicher oder sozialer Akzentuierung vor (2010, S. 295).

Bezüglich der Systematisierung nehmen wir die Anregung aus der vorliegenden Literatur auf, zwischen Arbeits- und Veranstaltungsformen unterschiedlicher *Größe* und *Steuerung* zu unterscheiden. Während im „Selbststudium“ (Götz und Häfner 1994, S. 86) die *Adressaten* auf sich selbst gestellt sind und ein autodidaktisches Setting in den formalen und informellen Rahmen übertragen, ist in einem „Training“ (Große Boes und Kaseric 2006, S. 14) ein *Verfahrensverwalter* federführend tätig. Wir möchten die Unterteilung der Arbeits- und Veranstaltungsformen in solche, in denen der pädagogisch Tätige als Taktgeber auftritt (bspw. im Frontalunterricht), im Vergleich zu solchen, in denen der pädagogisch Andere als Verfahrensverwalter fungiert, wie z. B. in selbstorganisierten Lernprozessen, ergänzen. Zur letztgenannten Dimension zählen wir auch die folgenden Varianten: „Praktikum“ (Götz und Häfner 1994, S. 80), „Arbeitsgruppe“ (Blom 1994, S. 17) oder Gruppenarbeiten wie die „Lerngruppe“ (Schäffter 1994, S. 68), „Team (Übung)“ (Große Boes und Kaseric 2006, S. 33), „Fallarbeit“ (Knoll 1999, S. 154), und „Computerbasiertes Lernen“ (Siebert 2004, S. 28).

Die pädagogische Fachkraft ist in der Mehrheit der Arbeits- und Veranstaltungsformen strukturierend. Hierunter zählen „Klassenzimmerunterricht“ (Götz und Häfner 1994, S. 81), „Workshop“ (Götz und Häfner 1994, S. 80), „Training“ (Große Boes und Kaseric 2006, S. 14), „Seminar“ (Götz und Häfner 1994, S. 80), „Schreibwerkstatt“ (Siebert 2004, S. 22), „Zukunftswerkstatt“ (ebd., S. 27), „Lernberatung“ (ebd., S. 34) oder auch die „Aufsuchende Bildungsarbeit“ (ebd., S. 17). Der Vortrag (vgl. Knoll 1999, S. 80) wird am augenfälligsten von der pädagogisch handelnden Person strukturiert. Als interessant erachten wir, dass die *Arbeitsgemeinschaft* als klassische und historisch verankerte Arbeits- und Veranstaltungsform in der Datenbasis nicht prominent erwähnt wird.

Arbeits- und Veranstaltungsformen können unterschiedliche *Gruppengrößen* annehmen (Verhältnis der Zahl der Besucher zur räumlichen Infrastruktur). Während Tagungen, Versammlungen oder Konferenzen vergleichsweise große pädagogische Settings sind und bestimmte architektonische und andere räumliche Arrangements voraussetzen, sind das Selbststudium, die Lernberatung oder auch die aufsuchende Bildungsarbeit Formen, deren Frequentierung von nur wenigen Beteiligten abhängt. Eher wenig erforscht ist die Frage, welche obligatorischen und welche fakultativen Elemente solche bekannten Arbeits- und Veranstaltungsformen, wie etwa Seminare, Trainings, Teamschulungen, Vorträge und Schreibwerkstätten, aufweisen.

Resümierend können wir festhalten, dass Arbeits- und Veranstaltungsformen ein stabiles Element innerhalb der Technologien darstellen. Sie können programmübergreifend angewendet und von den pädagogisch Tätigen mit einem spezifischen Profil aus Methoden und Medien versehen werden, wobei deren Kombination entscheidend sein dürfte. Hierfür bedarf es architektonischer und technischer Voraussetzungen, je nach Größe und Steuerungsbedarf. Die Steuerung kann sowohl durch die pädagogisch Tätigen als auch durch die pädagogisch Anderen erfolgen. Die Größe der Arbeits- und Veranstaltungsformen (Verhältnis Besucherzahl zu räumlicher Infrastruktur) ist maßgeblich dafür, welcher Organisationsaufwand benötigt wird. Interessant ist die Beobachtung, dass aus einer organisationsübergreifenden Perspektive unzählige Varianten der Benennung für ähnliche Arbeits- und Veranstaltungsformen existieren. Doch der dadurch entstehende Eindruck der Beliebigkeit der Benennung in der Literatur verhält sich gegenläufig zu der Praktik verschiedener Organisationen, in denen diverse Arbeits- und Veranstaltungsformen lediglich eine Bezeichnung bekommen, wie z. B. die Bezeichnung Kurs in der Volkshochschule.

### Methoden

Methoden definieren wir in einem ersten Zugriff als in der Regel schriftlich fixierte Verfahrens- und Vorgehensweisen, um im didaktischen Dreieck (Teilnehmerinnen und Teilnehmer – Pädagoginnen und Pädagogen – Thema/Sache/Vermittlungsgegenstand) die Unwägbarkeiten zwischen Vermittlungs- und Aneignungsprozessen zu minimieren und der Interaktion zwischen den drei Seiten eine zielgerichtete und produktive Dynamik zu geben. Über diese Technologie existiert eindeutig die meiste Literatur. In pädagogischen Ausbildungen nehmen die Methoden eine strategisch wichtige Stellung ein. Der Begriff griechischen Ursprungs definiert ein zielgerichtetes Vorgehen (vgl. Rassiller 2012, S. 383). Siebert (2004) formuliert folgendermaßen:

Methoden sind Wege, die zu Lernzielen führen. Sie sollen Lehr-Lern-Situationen gestalten, die in der außerschulischen Bildung außerordentlich vielfältig sind. Methoden sind dabei ausdrücklich keine technologischen Verfahren, die wie Schablonen eingesetzt werden können, sondern sie müssen von Veranstaltung zu Veranstaltung immer wieder neu abgestimmt werden: auf die spezifischen Situationen und Bedürfnisse immer wieder neuer Adressat/inn/en, auf wechselnde Inhalte und Lernziele sowie auf unterschiedliche Veranstaltungsformate. (ebd., S. 7)

Knoll (1999) definiert die Methode als „helfende Verfahrensweise“ (ebd., S. 12), um

- „vorhandenes Interesse zu verstärken,
- Informationen wirklich „ankommen“ zu lassen,
- eigene Einfälle und Ideen zu fördern,
- das wechselseitige Gespräch zu fördern,
- die Auseinandersetzung mit verschiedenen Themen anzuregen,
- gemeinsames Tun in Gang zu bringen.“ (ebd., S. 12)

Es gibt zahlreiche Systematisierungsversuche, um die Methodenvielfalt überschaubarer zu gestalten, wie z. B. bei Völzke in „Anfangs‑, Durchführungs‑, Auswertungs- und Abschlussmethoden“ (2012, S. 384). Götz und Häfner unterteilen in offene, projektorientierte, erlebnisorientierte, prozessorientierte, problemorientierte, objektorientierte Methoden (vgl. 1994, S. 112 ff.) sowie in Aktions- und Sozialformen (vgl. ebd., S. 43). Sie bevorzugen die Definition, dass Methoden „Wegfragen“/„Verfahrensweisen im Unterricht“ sind, also das Vorgehen nach dem Unterricht organisiert ist und Lehren und Lernen durchgeführt werden (ebd., S. 111). Eine ähnliche Systematisierung nimmt Siebert (2004) vor, wobei er in Aktionsformen, Sozialformen, Organisationsformen, Medien, Formen der Verständigung sowie Wirkungskontrolle und Prüfungen (ebd., S. 9) differenziert. Aufschlussreich ist, dass hier Medien in den Methodenbegriff inkludiert werden, weil dies die Flexibilität und die Varianz der in der Methodenliteratur üblichen Sprache unterstreicht. Geißler (1999) weist auf die didaktischen Ebenen hin, die bei einer Methodenentscheidung berücksichtigt werden sollten: Inhaltsebene, Gruppenebene und Einzelsubjektebene (vgl. ebd., S. 31). Siebert vermischt zwar nach unserer Definition Methoden und Arbeits- und Veranstaltungsformen, genauso wie Schäffter (vgl. 1994, S. 68), allerdings stimmt er zu, dass Methoden in dem Sinne keine rein technischen „Verfahren“ sind, die man wie „Schablonen“ nutzen kann, sondern sie eher kontextbezogen zu betrachten sind (Siebert 2004, S. 7). Die unterschiedlichen Systematisierungen sind der Vielzahl an Methoden und Anwendungskontexten und den Perspektiven der Betrachtenden geschuldet. Mal sind es eher mikrodidaktische Anleitungen zur konkreten Durchführung, mal versuchen sie die Rahmenfaktoren wie Teilnehmer, Ziele, Inhalt oder Institution (Knoll 1999) in ein nachvollziehbares Schema zu integrieren.

**Figure 3:**
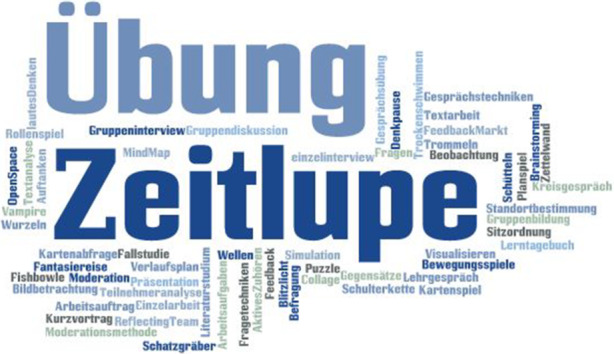

In unserer Datenbasis haben wir Methoden (Abb. 3) zu den folgenden Systematiken vorgefunden: spielerische Methoden, erkenntnisgenerierende Methoden, Präsentationsmethoden, Vermittlungs‑/Durchführungsmethoden, aktivierende Methoden und Evaluationsmethoden. Vereinzelt tauchen Methoden im Material auf, die auch zwei Clustern zugeordnet werden können. Generell gilt: Methoden sind situativ nutzbar, sprich variabel im jeweiligen Kontext anwendbar. Ihr Gelingen ist maßgeblich vom professionellen Können und der Haltung der Anwendenden sowie von der Kooperationsbereitschaft der Teilnehmenden abhängig. Die allermeisten Methoden sind unabhängig von der lernraumgebenden Organisation anwendbar und nicht gegenstands- und/oder raumgebunden. Sie können flexibel in verschiedenen Kontexten eingesetzt werden, jenseits vom Programm sowie der Veranstaltungs- und Arbeitsform.

In einem früheren Beitrag (vgl. Nittel et al. 2020, S. 389) haben wir noch Durchführungs‑, Delegations- und Evaluationsmethoden differenziert. Nach Durchsicht der hier vorliegenden Datenbasis möchten wir diese Clusterung verfeinern. Die *Vermittlungs‑/Durchführungsmethoden* nehmen den größten Raum in der Methodenfamilie ein. Zu ihnen gehören „Gesprächs- und Fragetechniken“ (Große Boes und Kaseric 2006, S. 18) und Gruppengesprächsmethoden wie „Fishbowle“ (Knoll 1999, S. 169), „Rundgespräche“ (ebd., S. 157), „Plenumsdiskussionen“ (ebd., S. 17), „Brainstorming“ (Klebert et al. 1998, S. 157), „Rollenspiel“ (Knoll 1999, S. 188), „Reflecting Team“ (ebd., S. 84) oder „Blitzlicht“ (ebd., S. 80). Weiterhin zählen zu den Vermittlungs‑/Durchführungsmethoden auch anleitende Methoden wie „Übung“ (Schäffter 1994, S. 69), „Arbeitsaufgaben“ (ebd., S. 69), „Fragen“ (Schulze-Kruschke und Paschko 2019, S. 113), „lautes Denken“ (ebd., S. 111) oder „stimulierende Hinweise“ (Frey 1995, S. 76). Auch „Einzelarbeit“ (Knoll 1999, S. 145), „Textarbeit“ (ebd., S. 147) oder „Kartenfrage“ (Klebert et al. 1998, S. 159) sind Vermittlungs‑/Durchführungsmethoden, ebenso wie Gruppensteuerungsmethoden wie beispielsweise „Vorstellungsrunde“ (ebd., S. 163), „biografisches Kennenlernen“ (Siebert 2004, S. 53) und „Gruppenbildung“ (Knoll 1999, S. 204).

Unter *erkenntnisgenerierenden* Methoden verstehen wir solche, durch die im Rahmen von Lehr-Lern-Kontexten sowohl die pädagogisch Anderen als auch die pädagogisch Tätigen einen Wissenszuwachs erlangen können, ohne dass es um die Vermittlung eines festen Wissenskanons geht. Dies können z. B. „Gruppen- oder Einzelinterview“ (Götz und Häfner 1994, S. 57), „Befragung“ (Edelmann und Möller 1976, S. 79) und „Beobachtung“ (Götz und Tschacher 1995, S. 26), „Teilnehmeranalyse“ (Knoll 1999, S. 43), „Fragen“ (Frey 1995, S. 76) oder auch „Textanalyse“ (Edelmann und Möller 1976, S. 80) sein.

Die *Präsentationsmethoden* scheinen für die Leserin und den Leser wahrscheinlich die alltäglichsten zu sein: „Vortrag“ (Geißler 1999, S. 31), „Kurzreferat“ (Knoll 1999, S. 129), „Podiumsdiskussion“ (ebd., S. 137), „Mind Map“ (Siebert 2004, S. 69), „Markt der Möglichkeiten“ (ebd., S. 58) oder auch „Lehrgespräch“ (Knoll 1999, S. 143) sind allgegenwärtige didaktische Arrangements.

Einen vergleichbar großen Raum im Methodenspektrum nehmen die* Evaluationsmethoden* ein: „Feedback“ (Siebert 2004, S. 81), „Befragung“ (Edelmann und Möller 1976, S. 79), „Lernkontrolle“ (Schäffter 1994, S. 69) oder auch die beliebte „Punkteabfrage“ (Klebert et al. 1998, S. 162) sowie „Entwicklungsgespräche“ (Götz und Häfner 1994, S. 171) gehören in dieses Cluster.

Zwischen all diesen Methoden befindet sich ein spezieller Typus der kontextbezogenen Methoden: die *spielerischen Methoden* oder *Spiele*, die in allen anderen oben erwähnten Subkategorien zu finden sind. Hier finden sich in der Literaturbasis u. a. „Stille Post Variation“ (Große Boes und Kaseric 2006, S. 20), „Kartenspiel“ (ebd., S. 56), „Knoten“ (Vopel 1994, S. 84), „Schatzgräber“ (ebd., S. 87) oder „Schlangengrube“ (ebd., S. 93).

Die aktivierenden Methoden sind eine besondere Art. Sie folgen zwar auch den oben erwähnten pädagogischen Grundvarianten, sind jedoch nur in ihrem besonderen Kontext nachvollziehbar, wie beispielsweise „Auftanken“ (Vopel 1994, S. 50), „Ärger loswerden“ (ebd., S. 39), „progressive Entspannung“ (ebd., S. 24), „heilender Atem“ (ebd., S. 29), „Entgiftung“ (ebd., S. 40) oder auch „Kraft schöpfen“ (ebd., S. 42).

Wir können sagen, dass die Methode diejenige Technologieform darstellt, die quantitativ am häufigsten in der von uns ausgewerteten Literatur repräsentiert ist. Für diese gilt, dass ihr Einsatz in der Verantwortung der pädagogischen Fachkraft liegt (gilt nicht ausschließlich für Evaluationsmethoden wie z. B. Feedbackbögen, die ggf. von der jeweiligen Organisation vorgegeben sind) und sie damit unabhängig von der rahmengebenden Organisation sowie dem Programm und den Arbeits- und Veranstaltungsformen ist. Methoden werden dem pädagogisch Tätigen nicht vorgegeben, er wählt sie in Anlehnung an den sozialen und inhaltlichen Kontext aus. Deshalb sind sie spontan und flexibel in ihrer Anwendung. Ihr Gelingen dürfte stark von der Professionalität des Anwendenden abhängig sein. Sie sind schriftlich kodifiziert, zeitlich begrenzt und von räumlichen Gegebenheiten meistens losgelöst. Eine Arbeits- und Veranstaltungsform kann mehrere Methoden umfassen. Methoden können in Präsentations‑, Vermittlungs‑, Durchführungs- und Evaluationsmethoden differenziert werden, wobei sich der Modus in eine spielerische und eine erkenntnisgenerierende Variante unterteilen lässt. Auf der Basis unseres Materials ist festzuhalten, dass die Vermittlungs- und Durchführungsmethoden besonders häufig präsent zu sein scheinen. Dahinter rangieren die erkenntnisgenerierenden Methoden, die Präsentationsmethoden oder die Evaluationsmethoden. Das Cluster der spielerischen und aktivierenden Methoden beinhaltet stark kontextbezogene Methoden, die nur in ihrem situativen Rahmen nachvollziehbar sind.

### Medien

Der Medienbegriff ist aktuell allgegenwärtig. Im Alltagsgebrauch verstehen wir unter Medien meist technikbasierte Medien. Dabei umfasst der Begriff, laut seinem lateinischen Ursprung, ein vermittelndes Element (Friedrichs 2012, S. 347), bietet eine Vielzahl an visuellen, haptischen, auditiven (vgl. Nittel et al. 2020, S. 390) oder auch audiovisuellen Möglichkeiten (Kipper 2014, S. 163) und kann auch Mimik oder Sprache und immaterielle Vermittlungsmedien umfassen (Friedrichs 2012, S. 347 f.). Das Medium existiert unabhängig von den anderen pädagogischen Technologien. Es wird zweckgebunden von pädagogischen Rollenträgerinnen und Rollenträgern und in Abhängigkeit des jeweiligen Wissens und Könnens ausgewählt (vgl. Nittel et al. 2020, S. 389). In der pädagogisch-didaktischen Perspektive werden Medien zur Zielerreichung in Lehr-Lern-Kontexten eingesetzt. Sie ergänzen damit Methoden und stehen selten im Fokus des Lernsettings. Eine Ausnahme bilden Arbeits- und Veranstaltungsformen z. B. innerhalb einer Trainerausbildung, in der explizit Medienkompetenz trainiert wird. Ein anderer Grund für die Fokussierung auf Medien könnte die Aktualität des jeweiligen Mediums sein. Solch ein Medieneinsatz wird dann wegen seiner Beliebtheit und nicht aufgrund seiner pädagogischen Relevanz gewählt.

Der Einsatz von Medien im pädagogischen Setting ist oftmals situativ innerhalb der Entwicklung der Arbeits- und Veranstaltungsform, allerdings nicht strikt an sie gekoppelt und dient zur Unterstützung oder Ausgestaltung einer Methode, die meist ebenso situativ verwendet wird. Ist die Medienauswahl erzwungen, nicht am pädagogischen Setting ausgerichtet und dominiert den Lernraum, kann sie sich kontraproduktiv auf das Lernziel auswirken.

**Figure 4:**
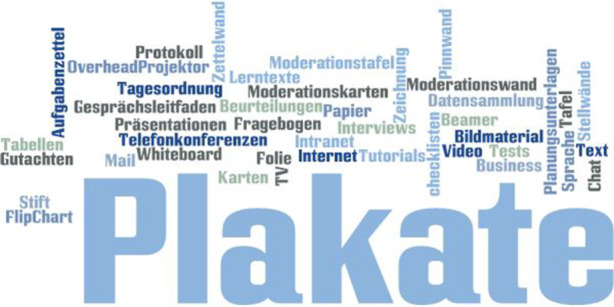

In Anlehnung an Heimann et al. (1977) begreifen Götz und Häfner Medien als Mittel, die dazu dienen sich über den Unterrichtsinhalt auszutauschen (vgl. Götz und Häfner 1994, S. 43) und die damit sowohl Personen als auch Sachen oder Ereignisse (vgl. ebd., S. 127) einschließen. Aus didaktischen Gesichtspunkten empfehlen sie eine Systematisierung nach inhaltlicher Qualität und didaktischem Ort (vgl. ebd., S. 128). Götz und Tschacher (1995) nennen explizit unter der Bezeichnung als Medium verschiedene Materialien (ebd., S. 19), die wir so auch zuordnen würden. Der Umstand, dass die Autoren ansonsten gegenüber unseren Kategoriensystem eine andere Systematik wählen, nehmen wir zur Kenntnis, ohne dies in irgendeiner Weise bewerten zu wollen – fußt doch der Entdeckungszusammenhang der pädagogischen Technologien auf der Analyse beruflicher Selbstbeschreibungen unterschiedlicher Gruppen von Pädagoginnen und Pädagogen über ihr faktisches Tun und nicht auf der Analyse didaktischer Literatur. In Abb. 4 finden sich Beispiele aus der von uns untersuchten Literatur.

In dieser Auswahl überwiegen *visuelle Medien*. Sie können konzeptioneller Natur sein, wie beispielsweise „Planungsunterlagen“ (Götz und Häfner 1994, S. 57), Zeichnungen (vgl. Große Boes und Kaseric 2006, S. 21), Protokolle (vgl. Klebert et al. 1998, S. 151), „Aufgabenbeschreibungen“ (Götz und Häfner 1994, S. 57), Lerntexte (vgl. Arzberger und Brehm 1995) und „Texte“ (Götz und Häfner 1994, S. 135) allgemein, „Tabellen“ (Blom 1994, S. 31), „Tagesordnung“ (ebd., S. 95), „Bildmaterial“ (Knoll 1999, S. 41), „Folien“ (Siebert 2004, S. 67), „Checklisten“ (Blom 1994, S. 120), „Bücher“ (Götz und Tschacher. 1995, S. 19), „Fragebögen“ (ebd., S. 19), „Schulungsunterlagen“ (ebd., S. 43) oder auch ein „Interviewleitfaden“ (ebd., S. 19) und eine „Zettelwand“ (Knoll 1999, S. 81). Überwiegend *computerbasierte visuelle Medien* sind „E-Mail“ (Wölker und Götz 2000, S. 14), „Chat“ (ebd., S. 32) und „Newsgroup“ (ebd., S. 44), eine Form des elektronischen schwarzen Brettes. Es ist allerdings auch möglich, dass auch von den oben erwähnten visuellen Medien einige ebenfalls in elektronischer Form vorliegen. Medien können, müssen aber nicht zwangsläufig Bezüge zu den modernen Techniken des Internets oder zur Massenkommunikation aufweisen. *Auditive Medien* sind in den seltensten Fällen in der Datenbasis vorhanden. Lediglich „Sprache“ (Götz und Häfner 1994, S. 135) und „Telefonkonferenzen“ (Busch und Götz 2000, S. 44) werden als Reinformen genannt. Die *audiovisuelle Medienkombination* ist dagegen öfter vertreten. Hier werden die folgenden genannt: „Videokonferenzsystem“ (ebd., S. 13), „Internet“ (ebd., S. 13), „Intranet“ (ebd., S. 16) und „Extranet“ (ebd., S. 17), allerdings auch „Präsentationen“ (Götz und Häfner 1994, S. 128), „Film“ (Knoll 1999, S. 16) oder „Business TV“ (Wölker und Götz 2000, S. 22). Auffällig ist, dass frühe klassische audiovisuelle Medien wie die Videokassette nicht genannt und hier nur computerbasierte Varianten aufgeführt werden.

*Habtische Medien*, wie „Whiteboard“ (Wölker und Götz 2000, S. 37), „Moderationswand“ (Große Boes und Kaseric 2006, S. 32), „Moderationskarten“ (ebd., S. 40), „Papier & Stift“ (ebd., S. 41), „Plakate“ (Klebert et al. 1998, S. 27), „Karten“ (ebd., S. 27), „Stellwände“ (ebd., S. 27), „Moderationstafel/Pinnwand“ (ebd., S. 160) finden sich im Vergleich zu den visuellen Medien ebenfalls weniger.

Dass haptische und auditive Medien in der analysierten Literatur eher weniger vertreten sind, kann mit der Fokussierung auf die erwachsenenbildnerische Datenbasis erklärt werden. Würden wir auf Material aus dem Elementar- oder Schulbereich schauen, könnte wahrscheinlich ein anderes Bild entstehen.

Die pädagogisch Tätigen wählen Medien zweckgebunden, meistens flexibel situativ anhand ihres Erfahrungsspektrums und den räumlichen Gegebenheiten aus. Einerseits hat die Organisation somit in der Regel wenig Einfluss auf den Einsatz und damit die mediengestützte Vermittlungssituation, andererseits stellt die Organisatin den Ort der pädagogischen Handlung zur Verfügung und beeinflusst somit doch die Medienauswahl. Medien stehen selten im Fokus des Lehr-Lern-Settings, wenn sie jedoch übermächtig werden, kann ihr pädagogischer Effekt verloren gehen. Anhand der Datenbasis differenzieren wir in visuelle, auditive, audiovisuelle und haptische Medien. Bis vor kurzem überwogen haptische Medien, durch die Entwicklung neuer Technologien, nehmen audiovisuelle Medien immer mehr Raum ein. Interessanterweise fand die Tafel, als das ursprünglichste pädagogische Medium in der analysierten Methodenliteratur keine Erwähnung, obwohl aus ihr vielfältige andere Medien entsprungen sind (Overhead, Whiteboard, Pinnwand, etc.).

### Zwischenfazit der Dokumentenanalyse

**Figure 5:**
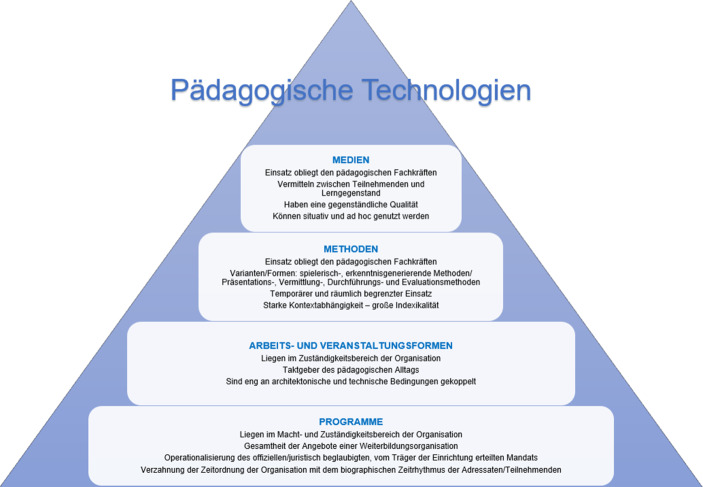

Die wesentlichen Merkmale pädagogischer Technologien können wie in Abb. 5 zusammengefasst werden.

Während die zwei zuerst erläuterten Technologien im Zuständigkeitsbereich der Weiterbildungsorganisationen liegen, sind die Methoden und Medien im unmittelbaren Einflussbereich des haupt-, neben- und freiberuflichen Personals zu verorten. Die Abbildung zeigt, dass jede Technologie für sich stehende spezifische Merkmale aufweist, die sie zwar klar von anderen abgrenzt, allerdings zugleich die Kontextbezogenheit unterstreicht. Das Gefüge der Technologien ist hierarchisch aufgebaut und folgt dem Modell der konditionellen Programmierung (Luhmann 1999, S. 98): Das Programm stellt die Basis für alle anderen Technologien dar und kann aus mehreren Arbeits- und Veranstaltungsformen bestehen, diese wiederum beherbergen eine Vielzahl von Methoden. Methoden werden durch Medien flankiert. So gesehen sind die pädagogischen Technologien ineinander geschachtelt und die jeweils rahmende gibt den Kontext für die darin liegende Technologie vor.

Wie die Literaturbasis gezeigt hat, werden die pädagogischen Technologien zumeist kontextbezogen bestimmt, und das verschafft der pädagogischen Praxis ein hohes Maß an Flexibilität. Der Verzicht an Trennschärfe fällt vor allem im Verhältnis zwischen den Arbeits- und Veranstaltungsformen und den Methoden auf. Unter Kontextbezogenheit verstehen wir, dass die Grenze der Arbeits- und Veranstaltungsform zur Methode fließend ist. Unter bestimmten Bedingungen können diese umgewidmet werden. So kann es z. B. bei der Kleingruppenarbeit vorkommen, dass diese als eine Arbeits- und Veranstaltungsform innerhalb eines Programmes fungiert oder aber als Methode genannt wird. Diesbezüglich ist folgendes Richtmaß für uns orientierungsrelevant: Eine Arbeits- und Veranstaltungsform tritt in der Regel immer im Singular auf. Im Rahmen einer Vortragsreihe in einem naturwissenschaftlichen Museum etwa werden Vorträge gehalten. Mit dieser Arbeits- und Veranstaltungsform soll ein bestimmter Zweck erfüllt werden, z. B. die Popularisierung von wissenschaftlichem Wissen. Findet dann – wider Erwarten – tatsächlich ein Wechsel zunächst vom Vortrag zur Kleingruppenarbeit und dann schließlich zur Museumsführung statt, so dienen die Kleingruppenarbeit und die Museumsführung als Methode, weil sie einerseits im Plural auftreten und andererseits mit ihnen kurzfristig und flexibel bestimmte Ziele innerhalb der rahmenden Arbeits- und Veranstaltungsform erreicht werden sollen. Die übergeordnete Arbeits- und Veranstaltungsform tritt in der Regel im Singular auf und führt einen ganz bestimmten pädagogischen Zweck mit sich. Demgegenüber kann die darunter rangierende Technologie der Methode auch als Vielheit genutzt werden, um Teilziele innerhalb des vorgegebenen Zwecks zu erreichen. Die umgekehrte Konstellation, nämlich dass Arbeits- und Veranstaltungsformen in einer Mischform angeboten werden, während die Methode exklusiv, d. h. nur im Singular auftritt, ist in dem hier vorgeschlagenen Ordnungsschema unvereinbar. Die Anwendungslogik von Methoden ist flexibel und situativ auf die Erreichung eines kurzfristigen didaktischen Zieles ausgerichtet, während Arbeits- und Veranstaltungsformen im Vergleich dazu sich durch eine gewisse Trägheit auszeichnen. Sie folgen einem übergeordneten Zweck; allein dieser invariante Telos schließt einen häufigen Wechsel aus.

Unter dem Eindruck der COVID-19-Pandemie in den Jahren 2020 und 2021 war verstärkt zu beobachten, dass die Programme ihre ordnungsstiftende Struktur einbüßten und diese an die technikgestützten Medien abgaben. Medien waren und sind seit dieser Zeit übermächtig. Sie scheinen pädagogische Situationen immer mehr zu dominieren. Rahmende Prozesse und Verbindlichkeiten, die normalerweise durch Programme geschaffen werden, verlieren offenbar ihre Wichtigkeit. So übernehmen Medien die Funktion, die eigentlich den Programmen zugeschrieben wird: viele Dimensionen des pädagogischen Handelns richten sich an ihnen aus, anstatt dass Medien in ihrer ursprünglichen Funktion die pädagogische Intention unterstützen. Das Medium ersetzt sogar im Fall des Online-Lehrens den räumlichen Bezug der Arbeits- und Veranstaltungsform. Eine Diskussion der Konsequenzen dieser Entwicklung auf pädagogisches Handeln nach der COVID-19-Pandemie steht noch aus. Tendenzen dieser Entwicklung zeichnen sich allerdings schon seit Jahren ab: So führt die Modularisierung von Programmen etwa dazu, dass die rahmengebenden Programme ihre Funktion an die Arbeits- und Veranstaltungsformen abgeben, wobei die rasante technische Entwicklung die Dominanz der Medien mit Sicherheit noch verstärkt haben dürfte.

## Pädagogische Kernaktivitäten

Wie der Ausdruck bereits andeutet, handelt es sich bei Kernaktivitäten um ein Set an symptomatischen und typischen Handlungen mit einem gewissen Wiedererkennungswert. Kernaktivitäten stellen elementare pädagogische Routinepraktiken dar, die prinzipiell von allen in der Erwachsenenbildung tätigen pädagogischen Fachkräften im Format eines einfachen Sprechaktes ausgeführt werden können, und zwar gleichgültig, ob das beiläufig oder gezielt – mit viel oder wenig Professionalität – geschieht. Die Kernaktivitäten des *Organisierens*, des *Unterrichtens*, des *Begleitens*, des *Beratens* und des *Sanktionierens* sind charakteristisch für das pädagogisch organisierte System des lebenslangen Lernens (Nittel et al. 2020; Nittel 2021). Entscheidend dabei ist, dass dem Konzept der Kernaktivitäten die Substantivierung von Verben zugrunde liegt. In diesem Fall ist also von *dem* Begleiten, *dem* Organisieren, *dem* Beraten, *dem* Unterrichten sowie *dem* Sanktionieren die Rede. Mit dieser Spezifizierung soll gewährleistet werden, dass die Kernaktivitäten nicht mit ihren Institutionalisierungsformen verwechselt werden: Die Begleit*ung *als aufwendige Inklusionsmaßnahme in der Weiterbildungsarbeit mit behinderten Menschen darf folglich nicht mit dem Begleiten als ad hoc Handlung verwechselt werden. Und auch zwischen dem punktuellen Organisieren des eigenen Alltags und der Organisa*tion* als zweckrational agierende soziale Einheit gibt es gewaltige Unterschiede. Zwischen dem situativen Beraten zwischen Tür und Angel und der Praxis einer Weiterbildungsberatungsstelle liegen ebenso Welten, wie zwischen der Praktik des Unterrichtens im Sinne des Vermittelns einer Information und dem ritualisierten Unterricht im Kursbetrieb. Während spontane Gesten des Billigens oder Missbilligens im Alltag der Weiterbildung einen eher flüchtigen Charakter ohne langfristige Konsequenzen haben, können der Sanktion im Zuge einer aufwendigen beruflichen Wiedereingliederungsmaßnahme als offizieller, juristisch abgesicherter Schritt weitreichende Folgen innewohnen (z. B. der Entzug finanzieller Unterstützungsleistungen).

Beim Versuch, den flüchtigen und unsteten Charakter der eben angedeuteten Routinepraktiken im Weiterbildungsgeschehen zu verdeutlichen, werden wir nun den Alltag einer Fachbereichsleiterin an einer VHS (Volkshochschule) gedankenexperimentell simulieren und möglichst konkret beschreiben^10^. Würde man die ausgesprochen komplexe Praxis einer an einer VHS tätigen Fachbereichsleiterin (Monika Schmidt) für Sprachen unter Maßgabe der fünf Kernaktivitäten abtasten, so läge das folgende Szenario nahe an der Realität^11^:

### Organisieren

Als Mitarbeiterin einer städtischen VHS betritt Monika Schmidt wie jeden Tag gegen 8:30 Uhr die Einrichtung. Bevor sie die ersten Gespräche führt, vergewissert sie sich durch einen Blick auf ihren Terminkalender, was für den heutigen Tag ansteht: Neben einem Meeting mit allen anderen Fachbereichsleiterinnen und Fachbereichsleitern, bei dem es überwiegend um die Planung von Einzelveranstaltungen und kommender Sondersitzungen gehen wird, stehen auch die Vergütung von Dozierenden sowie einige Teilnehmerbeschwerden auf der Agenda. In einem folgenden Gespräch mit einigen Fachkräften aus dem administrativen Bereich werden danach die aktuellen Anmeldezahlen, das Anfragenmanagement sowie die Raumorganisation abgestimmt.

Dimensionen des Organisierens: Man organisiert die eigene Arbeit (reflexiver Bezug), leistet aber auch Hilfe für andere (transitiver Bezug).

Synonyme des Organisierens: Managen, Disponieren, Planen, Arrangieren, Entwerfen, Leiten, Führen.

### Unterrichten

Gegen 9:30 Uhr beantwortet Monika Schmidt eine Mail einer Kursleiterin, in welcher sie die Fragestellerin darüber unterrichtet, dass über die gestrige Informationsveranstaltung zu einem vielversprechenden Lehrwerk für Englischkurse, bei der die Kursleiterin abwesend war, ein Protokoll angefertigt werden wird. Um den Anforderungen einer Fachbereichsleiterin nach Transparenz für die Teilnehmenden, die Administration, die Leitung und die Kursleitenden gerecht zu werden, müssen alle, die an Bildung direkt oder indirekt beteiligt sind, regelmäßig und mit großer Sorgfalt auf den neuesten Stand gehalten – sprich: „unterrichtet“ werden.

Dimensionen des Unterrichtens: Die pädagogische Ziel- und Adressatengruppe, Vertretende der eigenen Organisation oder Akteure der institutionellen Umwelt werden unterrichtet.

Synonyme des Unterrichtens: Lehren, Unterweisen, Anleiten, Aufklären, Informieren, Vermitteln, Anlernen, Beibringen, Einweisen, Zeigen, Einarbeiten, Belehren, Instruieren, Erklären, Aufzeigen, Dozieren.

### Begleiten

Die Fachbereichsleiterin ist erst fünf Jahre an der hiesigen Volkshochschule. Längst ist ihr in dieser Zeit durch mehrere Erfahrungen klar geworden, dass es einige Kursleitende gibt, die nicht nur begleitet werden wollen, sondern aus Gründen der fachlichen Kompetenzentwicklung begleitet werden müssen. Begleiten bedeutet für Monika Schmidt also nicht nur *für die Kursleiterinnen und Kursleiter da zu sein*, sondern auch den betreffenden Personen in regelmäßigen Abständen Informationen über didaktisch-methodische Innovationen zu vermitteln und über Fortbildungsmöglichkeiten zu informieren. Ebenso fällt hierunter die Unterstützung einiger neuer Dozierenden bei der Konzepterstellung spezifischer Unterrichtskonzepte. Diese Kernaktivität hat aber noch eine andere Dimension: Monika Schmidt verfügt über präzise Informationen, welche der von ihr betreuten Kursleitenden ihrerseits Teilnehmende (Stammkundinnen und Stammkunden) über einen längeren Zeitraum begleiten. Unter dem Fokus der Professionalität weiß Monika Schmidt die Ambivalenz dieses Phänomens einzuschätzen: Einerseits stellt der langjährige Kontakt zu Kursleitenden ein Zeichen für die besondere Qualität der Veranstaltung dar; andererseits kann durch die damit u. U. verbundene Gruppenkohärenz die Integration von neuen Teilnehmenden erschwert werden und die Gefahr des *Klientifizierung* (Abhängigkeit) wachsen.

Dimensionen des Begleitens: Kurz-, mittel- und längerfristige Varianten des Begleitens sowie Da-Sein versus Akutintervention.

Synonyme des Begleitens: Helfen, Betreuen, Unterstützen.

### Beraten

Es ist kurz vor 13 Uhr, eigentlich steht die Mittagspause an, als die Verwaltungsmitarbeiterin anklopft und Monika Schmidt mitteilt, dass eine Besucherin eine Frage habe und sich weigere zu gehen. Schon nach dem Austausch von wenigen Worten mit der Besucherin merkt Frau Schmidt, dass hier ein lang aufgeschobenes Problem vorliegt. Sie ist sich sicher, dass dieses Anliegen nur in einem aufwendigeren Beratungsprozess bearbeitet werden kann. Die Besucherin, so stellt sich heraus, ist mit einer Entscheidungskrise konfrontiert: Sie steht vor der Wahl in Abhängigkeit von Karriereoptionen, entweder einen berufsorientierten oder eine kulturorientieren Englischkurs zu belegen. Monika Schmidt rät der Frau, eine Weiterbildungsberatung im eigenen Haus aufzusuchen, teilt ihr die Kontaktdaten der zuständigen Kollegin mit und stuft das Anliegen als komplex und anspruchsvoll ein, so dass dafür eine volle Beratungsstunde eingeplant werden soll.

Dimensionen des Beratens: Individuelle und kollektive Beratungsfälle.

Synonyme des Beratens: Tipps geben, Erörtern und Abwägen, Abraten und Zuraten, offen Bedenken formulieren, zum Nachdenken anregen.

### Sanktionieren

Monika Schmidt hat durch ihre eigene Praxis als Kursleiterin kurz nach ihrem Studium die Erkenntnis gewonnen, wie wichtig eine gute Feedback-Kultur in den Kursen der Erwachsenenbildung ist. Sie hat ein sicheres Gespür dafür, dass manche Teilnehmende schmerzhafte Schulerfahrungen mit sich herumtragen und Zuspruch enorm wichtig ist. Je länger sie an der VHS ist, desto bewusster wird ihr auch, dass es manchmal Situationen gibt, in denen auch die andere Seite im Spektrum des Sanktionierens bedient werden muss. Damit ist das situative Missbilligen von Handlungsweisen gemeint. Es passiert in ihren Augen nicht sehr häufig, jedoch kommt es vor, dass Kursleitende mit einem Migrationshintergrund offen oder subtil beleidigt werden oder dass Teilnehmende bürgerliche Anstandsregeln verletzen. Da in solchen Situationen die soziale Ordnung einer Einrichtung der Erwachsenenbildung verletzt zu werden droht und da es auch um die Fürsorgepflicht als Fachbereichsleiterin gegenüber den Dozierenden geht, ist Monika Schmidt in solchen Situationen gefordert, als Repräsentantin der Organisation zu agieren. Derartige Ausnahmesituationen sind, ohne das Sanktionieren im Sinne des Missbilligens, kaum zu meistern, auch wenn Hausverbote und Verwarnungen das letzte Mittel darstellen mögen.

Dimensionen des Sanktionierens: Billigen und Missbilligen, positive und negative Sanktionen.

Synonyme des Sanktionierens: Gutheißen, Belobigen, Würdigen, Bestätigen, Zustimmen, Bejahen versus Verbieten, Bestrafen, Zustimmung verweigern.

Pädagogische Situationen als kleinste und grundlegendste Einheit des pädagogisch organisierten Systems des lebenslangen Lernens (vgl. Nittel et al. 2020) werden sowohl von Technologien überformt als auch durch Kernaktivitäten gespeist. Wir nehmen dabei bewusst in Kauf, dass hier Ebenen und Phänomene analytisch getrennt werden, die in der Realität wie in einem Knäuel eng miteinander verflochten sind. Um die Ordnung eines Knäuels zu erfassen, erscheint es aber manchmal geboten, die Fäden fein säuberlich zu sortieren, diese für sich zu betrachten, da man nur so die Ordnung hinter der vordergründigen Unordnung einer flüchtigen pädagogischen Situation verstehen kann.

## Die Relationierung pädagogischer Kernaktivitäten mit Technologien einerseits und Systemmerkmalen andererseits

Bereits zu Beginn haben wir darauf hingewiesen, dass in den vorliegenden Publikationen der komparativen pädagogischen Berufs- und Organisationsforschung das Verhältnis zwischen den situativen Kernaktivitäten und den davon abgehobenen Institutionalisierungsformen nicht befriedigend geklärt werden konnte. Wir haben zwecks Behebung dieses Desiderats die eingangs erwähnte abduktive Forschungsstrategie konsequent fortgesetzt, indem wir uns die pädagogischen Technologien in der Erwachsenenbildung im Medium einer Dokumentenanalyse ganz konkret vor Augen geführt haben, ebenso wie wir versucht haben, uns die pädagogischen Kernaktivitäten im Zuge des Eintauchens in den pädagogischen Alltag einer VHS-Mitarbeiterin – entlang ihrer Erfahrungen – zu vergegenwärtigen. Das Hin-und-Herspringen zwischen den beiden Ebenen evozierte den kognitiven Zugzwang, bisher nicht bedachte Verbindungen zwischen den beiden Aggregatebenen der Ordnung des Pädagogischen in Betracht zu ziehen. Mit dem hier ventilierten Gefüge an Kernaktivitäten und der Technologien haben wir Phänomene konstruiert, die zunächst einmal auch bei uns Verwirrung gestiftet haben: Wie sollen die Beobachtungen aus den Dokumenten mit den quasi-ethnographischen Impressionen verbunden werden? Wie passt die eine mit der anderen Forschungsperspektive zusammen? Die so gestaltete Inszenierung einer Erkenntniskrise hätte genauso gut scheitern können. Von entscheidender Bedeutung für die konstruktive Lösung war, dass die Technologien und die Kernaktivitäten anschaulich vor uns lagen und gleichzeitig der Zugzwang zur Abstraktion sehr massiv war. Forschung ist bekanntlich das Ausräumen von Zweifel durch das Finden neuer Regeln. Deren Generierung erfolgte aber nicht ad hoc, sondern prozesshaft, und zwar durch beständiges Räsonieren, Gedankenexperimente und das damit verbundene Verwerfen von Hypothesen (Fallibilismus) sowie durch die Aufstellung von neuen Erklärungsversuchen. Wir können nicht mit Bestimmtheit sagen, welche Impulse uns zur Lösung des Problems verholfen haben: War es die Beschäftigung mit dem systemtheoretischen Begriff „Reentry“, die Rezeption relationstheoretischer Ansätze oder war es eine blitzartige Erkenntnis beim Abgleich der beiden Materialtypen? Sicher ist nur, dass wir den Vorgang des Suchens nach einer erklärenden Hypothese abschließen und ein bislang unverständliches Phänomen verstehen konnten. Der hier angedeutete abduktive Erkenntnisprozess hat uns von der Vermutung Abstand nehmen lassen, dass alle Kernaktivitäten in ein und derselben Weise mit den Technologien verbunden sind. Am Ende des wissenschaftlichen Suchprozesses können wir die Feststellung formulieren, dass die fünf Kernaktivitäten zum einen in das Gefüge der Technologien und zum anderen über den Weg der Institutionalisierung in das Funktionssystem des lebenslangen Lernens gleichsam wieder eintreten. Momentan zeichnet sich mit Blick auf die hier ventilierten empirischen Impulse die folgenden Konstellationen ab:

- *Das Unterrichten und Beraten* *→* *Referenz* *→* *die Arbeits- und Veranstaltungsform Unterricht sowie Beratung*Sowohl das Unterrichten als auch das Beraten stellen höchst flüchtige Praktiken im pädagogischen Alltag dar, deren performativen Vollzugs sich die Fachkräfte keineswegs immer bewusst sind. Leicht nachvollziehbar ist, dass die Kernaktivitäten des Unterrichtens und des Beratens, wenn sie sich habituell verdichten, als komplementäre Institutionalisierungsformen, nämlich in Gestalt von *Unterricht* und *Beratung* als nahezu klassische Arbeits- und Veranstaltungsformen, den Technologien zuzuordnen sind. Der Unterricht stellt nicht nur im Schulsystem, sondern auch in anderen Segmenten des pädagogisch organisierten Systems des lebenslangen Lernens, eine prototypische Arbeits- und Veranstaltungsform von hoher Stabilität und einem großen Wiedererkennungswert dar. In den Universitäten ist diese Arbeits- und Veranstaltungsform beispielsweise ebenso anzutreffen wie in bestimmten Bereichen der Erwachsenenbildung. Die Beratung als Arbeits- und Veranstaltungsform weist, wie der Unterricht, ein hohes Niveau der Institutionalisierung auf: Beratungsstunden in der Schule, Ausdifferenzierung bestimmter fachgebundener Rollen (Beratungslehrer), räumlichen Arrangements (Beratungszimmer, Beratungszimmer) und spezielle Beratungseinrichtungen (Drogenberatung, Familienberatung usw.) (vgl. Gieseke und Nittel 2016).
- *Das Organisieren* *→* *Referenz* *→* *das Systemelement „Organisation“*
  Ganz anders verhält es sich mit den drei verbliebenen Kernaktivitäten, diese kommen nicht auf der Ebene der Technologien, sondern an anderer Stelle im pädagogisch organisierten System des lebenslangen Lernens zur Geltung. So vollzieht die Kernaktivität des Organisierens insofern eine gewaltige Metamorphose, als sie gleichsam in verdinglichter, geronnener und verdichteter Gestalt als (pädagogische) Organisation einem der drei Systemelementen zugeordnet werden kann. Gäbe es die Unterformen der Kernaktivität des Organisierens nicht (Verwalten, Planen, Dokumentieren und Disponieren), so verlöre die Organisation als soziale Einheit ihre handlungsschematische Erdung. Die Totalität der Erziehungs- und Bildungsorganisationen – das ist an einer anderen Stelle erläutert worden (Nittel 2017; Wahl et al. 2022) – stellt eines der drei Systemelemente im pädagogisch organisierten System des lebenslangen Lernens dar^12^. Die Institutionalisierungsform der Organisation auf der konstituierenden Ebene des Systems korrespondiert so gesehen mit der situativen Routinepraktik des Organisierens im pädagogischen Alltag: das „Kleine“ in der Handlungspraxis verschränkt sich mit einem systemischen Strukturmerkmal im „Großen“.
- *Das Begleiten und Sanktionieren* *→* *Referenz* *→* *die Systemfunktion: Begleitung versus Selektion*
  Das Begleiten und das Sanktionieren als die beiden verbliebenen Kernaktivitäten können primär dem Funktionszusammenhang des pädagogisch organisierten Systems des lebenslangen Lernens zugeordnet werden; nicht den Technologien und nicht den Systemelementen. Wir alle sind momentan Zeugen, wie sich der bislang dominante Operationsmodus der Vorbereitung des Erziehungs- und Bildungswesens in Richtung biographische Begleitung wandelt (Nittel und Meyer 2018). Das Begleiten ist im Mikrobereich der Interaktion (Begleitung im Alltag) ebenso präsent wie im Zuge der Inklusion von Gesellschaftsmitgliedern in pädagogische Organisationen über die gesamte Lebensspanne. Der zuletzt genannte Aspekt ist in der Erwachsenenbildung besonders evident, deckt doch die Weiterbildung das allergrößte Zeitvolumen im Lebenszyklus ab.
  Das positive und negative Sanktionieren kann der hinreichend vom Strukturfunktionalismus beschriebenen Selektionsfunktion des Erziehungs- und Bildungswesens zugeordnet werden. Obwohl große Teile der Erwachsenenbildung, besonders die allgemeine Weiterbildung, die kulturelle und die politische Bildung, offensichtlich nur eine schwache Selektionsfunktion besitzen, darf ihre Rolle bei der Reproduktion sozialer Ungleichheit nicht unterschätzt werden. Besonders klar tritt die Selektionsfunktion in der beruflichen Bildung und in der betrieblichen Bildung mit seiner engen Verknüpfung zum Personalwesen zutage.

Diese Überlegungen machen deutlich, dass mehrfache Relationierungen zwischen der situativen Ebene der Kernaktivitäten mit höher aggregierten Institutionalisierungsformen existieren. Das bedeutet: Die Kernaktivitäten in der Interaktionssphäre sind gerade nicht von einer separaten Mesoebene oder als isolierte Größen von der Makroebene zu trennen, sondern sie sind bei der Produktion und Reproduktion des Systems über den Mechanismus des Wiedereintritts untrennbar entweder mit einer Technologie (Unterricht und Beratung), einem Systemelement (Organisation) oder dem Funktionskreislauf des Systems (Begleitung, Sanktionierung) verbunden. Oder anders ausgedrückt: Die flüchtigen Momente im Alltagsgeschäft der Erwachsenenbildung, der auf den ersten Blick profane Routinebetrieb und die vordergründig chaotischen Anteile im pädagogischen Alltag – all diese interaktiven Phänomene sind in unterschiedlicher Weise an der Formierung stabiler institutioneller Strukturen beteiligt. Sie stellen sich als strategisch relevant heraus und bilden Muster sowie Ressourcen der Konstitution sozialer Ordnung. So gesehen erweisen sich die methodischen Schritte der Kontrastierung von Daten aus der Dokumentenanalyse mit den quasi-ethnographischen Impressionen einerseits und der gegenstandsbezogene Abgleich als durchaus anschlussfähig gegenüber neueren relationstheoretischen Arbeiten im Bereich der Erziehungswissenschaft (von Eschenbach und Schäffter 2021). Vom Standpunkt der komparativen pädagogischen Berufs- und Organisationsforschung steht diese Untersuchung nicht allein da, sondern schließt an Arbeiten über die Schule (Nittel 2021) und die Elementarpädagogik (Buschle und Nittel 2022) sowie grundlagentheoretische Texte (Nittel et al. 2020) an. Sie zeigt, dass das hier entfaltete Kategoriensystem in Gestalt der Technologien und Kernaktivitäten problemlos auf die Erwachsenenbildung übertragbar ist. Dieser Befund stellt als solcher bereits eine interessante Mitteilung dar, da er die Erwachsenenbildung damit keineswegs als Grenzfall innerhalb des pädagogisch organisierten Systems des lebenslangen Lernens ausweist. Darüber hinaus kann erstmalig eine systematische Verbindung zwischen Technologien und Kernaktivitäten aufgezeigt werden. Mit Blick auf die anfangs erwähnten Ansätze von Giesecke sowie Prange und Strobel-Eisele deutet sich – analog zu der methodologischen Prämisse der Abduktion – zum Schluss eine überraschende Gemeinsamkeit an: Während Prange und Strobel-Eisele primär auf das Leitkonzept der Erziehung fokussiert bleiben, rekurrieren sowohl Giesecke als auch wir auf Lernen als das eigentlich innovative Einheitskonzept (vgl. Nittel 2021). Aber alle drei – ansonsten höchst unterschiedlichen – Modelle verbindet an einem Punkt offenbar ein ungeahnter Konsens: Sie verzichten auf ein normativ überhöhtes Konzept der Bildung. Das allein sollte einer Disziplin, die sich selbst mit dem Etikett empirische *Bildungs*forschung zu schmücken pflegt, zu denken geben.

## Footnotes

1 Zu diesem Forschungsstil hat sich einer des Autorenteams an einer anderen Stelle mit Verweis auf die Grounded Theory ausführlicher geäußert (Nittel 2012) (Grundlegend zu dieser Methodologie vgl. Apel 1975, S. 187 f., 309 f.). In der von uns verwendeten abduktiven Forschungsstrategie wechselt der Vollzug primär induktiver oder deduktiver Schritte immer wieder ab, wobei das daraus entstehende Spannungsverhältnis für die überraschenden Erkenntnisse mitentscheidend ist. Darüber hinaus werden im Rahmen singulärer Forschungsaktivitäten (wie etwa in der vorliegenden Studie) gezielt kognitive Dissonanzen erzeugt, und zwar durch die bewusste Vermeidung starrer Vorannahmen; die möglichst lange Aufrechterhaltung von Offenheit; den Vergleich von Daten, die auf den ersten Blick gar nicht zu vergleichen sind und schließlich die Triangulation von Methoden, ohne vorab zu wissen, welcher Nutzen damit verbunden ist.

2 Pädagogische Erwerbsarbeit im System des lebenslangen Lernens. Berufliche Selbstbeschreibungen und wechselseitige Funktions- und Aufgabenzuschreibungen Aktenzeichen DFG: NI 668/6‑2; vgl. Nittel et al. (2014).

3 In dieser Untersuchung wird das berufliche Wissen und Handeln von 28 Expertinnen und Experten aus Personalentwicklung und Qualitätsmanagement auf seine pädagogischen Anteile analysiert. Auch Meyer (2017) hat in seiner Dissertation einen Beitrag zur Ausdifferenzierung dieses Ansatzes geleistet.

4 Zwei Argumente sprachen dafür, in der beruflichen Praxis breit rezipierte Publikationen zur didaktisch begründeten Nutzung von Methoden in der Erwachsenenbildung auszuwählen: Zum einen sind die Varianten der Technologien ursprünglich aus der Analyse beruflicher Selbstbeschreibungen über die pädagogische Alltagspraxis von Vertretern der betrieblichen Bildung (Kipper 2014) und den erwähnten PAELL-Daten gewonnen worden, sodass kein methodischer Zirkelschluss drohte. Und zum anderen weisen die von uns stark gemachten Dimensionen der Technologien – schon auf den ersten Blick – eine gewisse Affinität zur Sprache in der Methodenliteratur auf, was die Frage nach der Differenz der beiden Ebenen aufwirft.

5 An dieser Stelle muss darauf hingewiesen werden, dass wir hier den Begriff der Methodenliteratur der Feldsprache entnehmen und der Einfachheit halber beibehalten. In der Sprachregel der Autoren müsste es korrekt eigentlich *Literatur mit einer Affinität gegenüber pädagogischen Technologien* heißen.

6 Zum kontrastiven Vergleich sind vereinzelt auch Werke aus den angrenzenden Feldern Ausbildung und Schule in den Materialbestand integriert worden.

7 Eine Bestätigung über die Teilnahme an einem Programm gilt in unserem Kulturkreis als selbstverständlich und beglaubigt in schriftlicher Form das Durchlaufen des Programmes.

8 Die Tatsache, dass Programme in der Datenbasis eher weniger bearbeitet und die anderen Technologien unabhängig von ihnen diskutiert werden, sollte an anderer Stelle weiterführend betrachtet werden, vor allem vor dem Hintergrund der wechselseitigen Beeinflussung oder der Loslösung einzelner Technologien von anderen.

9 Fernab der klassisch explizit pädagogischen Erwachsenenbildung finden sich Programme auch in nicht primär pädagogischen Handlungsfeldern, u. a. in Wirtschaftsorganisationen, die bspw. durch Job Rotation, Führungskräftenachwuchs- oder Mentoringprogramme ganze Zugehörigkeitsphasen des jeweiligen Mitarbeiters begleiten. Neuberger (1991) zählt z. B. vielfältige Technologien organisationaler Kontexte auf. Es entsteht eine Verbindung zwischen wirtschaftlich orientiertem und pädagogischem Handeln. Andere Fachdisziplinen nutzen die Technologien auch im Rahmen pädagogischer Settings, jedoch ohne den Kontext zur Ursprungsdisziplin herzustellen.

10 Das vorliegende Beispiel einer Fachbereichsleiterin wurde mit Experten aus Volkshochschulen kommunikativ validiert, bevor es hier zum Einsatz gekommen ist.

11 Wir wählen hier den Weg der quasi-ethnographischen Impressionen primär aus darstellungstechnischen Gründen: Wie die bereits vorliegenden Teilstudien zeigen, sind die zur Verfügung stehenden Interviewdaten (vgl. Buschle und Nittel 2022; Nittel 2021) oder auch andere Quellen (Experteninterviews aus dem DFG Projekt Qualitätsentwicklung; z. B. die Portraits aus der Erwachsenenbildung in Nittel und Völzke 2002) ausgesprochen komplex. Deren Analyse würde den Rahmen dieses Beitrags sprengen. Die Impressionen ersetzen keineswegs eine Realanalyse; sie erfüllen hier vor allem die Funktion der Veranschaulichung.

12 Die soziale Welt der pädagogisch Tätigen (Gesamtheit der in einer Leistungsrolle tätigen pädagogischen Fachkräfte) bildet das zweite Element und die pädagogisch Anderen (Totalität aller Personen, die zur Ziel- und Adressatengruppe von Dienstleitungen auf dem Gebiet von Erziehung und Bildung zählen) das dritte.
